# Extreme Artificial Airglow Induced by HF Pumping Sporadic E Layer at the SURA Facility

**DOI:** 10.3390/s26092644

**Published:** 2026-04-24

**Authors:** Alexander Beletsky, Ivan Tkachev, Savely Grach, Alexey Shindin, Igor Nasyrov, Denis Kogogin, Valery Emeljanov, Yulia Legostaeva, Elena Tareeva, Sergey Moiseev, Roman Vasilyev

**Affiliations:** 1 Institute of Solar–Terrestrial Physics SB RAS, Lermotova Street, 126A, a/b 291, Irkutsk 664033, Russia; tid007@iszf.irk.ru (I.T.); roman_vasilyev@iszf.irk.ru (R.V.); 2 Radiophysics Faculty and Radiophysical Research Institute, Lobachevsky State University, Gagarina Avenue, 23, Nizhny Novgorod 603022, Russia; sgrach@rf.unn.ru (S.G.); shindin@rf.unn.ru (A.S.); legostaeva@rf.unn.ru (Y.L.); elena.trvsv@yandex.ru (E.T.); moiseev@rf.unn.ru (S.M.); 3 Institute of Physics, Kazan Federal University, Kremlevkaya Street, 16a, Kazan 420008, Russia; igor.nasyrov@kpfu.ru (I.N.); dkogogin@kpfu.ru (D.K.); evv960722@gmail.com (V.E.)

**Keywords:** ionosphere, sporadic E layer, artificial airglow, powerful radio wave, the SURA facility

## Abstract

The paper presents experimental data on the observation of artificial airglow of the ionosphere induced by HF radio wave pumping by the SURA heating facility during the presence of a blocking sporadic E layer of the ionosphere. Optical observations were carried out on 5 August 2024 using a three-channel photometer and CCD cameras with narrow-band filters. Emission of atomic oxygen at the wavelength λ = 557.7 nm (green line), as well as airglow close to the red line of atomic oxygen at λ = 630 nm and the band of molecular nitrogen ions $1NGN_{2}^{+}\left(0-0\right)$ at λ = 391.4 nm (blue band), were recorded. The induced emission intensity in the green line reached ∼270 R, larger than ever measured. Additional lower-intensity glow spots in the green line southwest and northeast of the main spot (∼12° from zenith), detected by the CCD camera, could be due to the side lobes of the SURA antenna pattern. The atypical behavior of the time course of the intensity in the red line with sharp fronts of increase and decrease may indicate the detection of emission lines of hydroxyl groups in the OH(9-3) and OH(5-0) bands, spectrally close to 630 nm. More detailed analysis of the results obtained and new similar experiments will lead to a deeper understanding of the processes occurring in the upper atmosphere/lower ionosphere during conditions of high solar activity.

## 1. Introduction

Enhancements of the night airglow at different wavelengths, e.g., at λ = 630 nm (transition $O^{1}D\to O{(}^{3}P)$, red line of the atomic oxygen, radiative lifetime τ=0.7 s [1]), λ = 557.7 nm ($O{(}^{1}S)\to O{(}^{1}D)$, green line of the atomic oxygen), and λ = 391.4 nm, λ = 427.8 nm (blue lines of the nitrogen ion, $\tau =10^{-6}\;$ s [1]), due to modification of the F-region ionosphere by powerful high-frequency (HF) radio waves, have been studied since the early 1970s [2,3,4,5,6,7,8,9]. Such an emission is considered to be evidence that the HF-modified electron distribution function is non-Maxwellian because a significant flux of suprathermal electrons is required to produce the artificial airglow. A suprathermal tail is known to develop as a result of the electron acceleration by pump-induced plasma waves.

At the SURA facility (Nizhny Novgorod, Russia), such studies have been conducted since 1983 [8]. Experiments were carried out with the participation of foreign scientists in the 1990s and 2000s [4,10], and regular observations have been provided since 2006 [11]. The brightness of artificial airglow depends on the conditions of the experiment, such as the pump wave frequency and power, altitude of the pump interaction with ionospheric plasma, geographic position of the heating facility, ionospheric critical frequency, etc. For example, at the HAARP facility in the red and green lines, respectively, the brightnesses can achieve 280 R and 50–70 R [12], while, at the Arecibo heating facility, the brightnesses in the same lines were 50–70 R and ∼5 R [13]. At the EISCAT heating facility brightnesses of ∼50–70 R in the red line, ∼10 R in the green line and ∼5 R in the blue line (427.8 nm, $1NGN_{2}^{+}\left(0-1\right)$) were measured [14]. These values were obtained for the airglow generated in the F region.

Apart from the experiments described in this study, enhancements of the green line emission during the development of $E_{s}$ layers have been observed only a few times: at the Arecibo heating facility in January 1998 [15] and at the SURA facility in September 2021 [16] and August 2023 [1]. In the former experiment, 55 R airglow was registered at λ = 557.7 nm. Also, for the first time, emission at 640–680 nm (first positive neutral molecular bands of $N_{2}$) was observed, and possible enhancement of the emission in the 710–760 nm range was mentioned. The brightness of the artificial airglow at 557.7 nm generated in the $E_{s}$ layer at the SURA facility reached several Rayleighs [1,16]. Also, at the SURA facility, $E_{s}$-related blue line artificial airglow at λ = 391.4 nm was revealed [1].

In this paper we report the results of the experiment performed at the SURA facility on 5 August 2024 when, over a long duration (about 1.5 h), the existence of a powerful sporadic E layer with a blocking frequency more than 9 MHz, extremely strong artificial airglow at 557.7 nm and noticeable enhancement of the airglow close to 391.4 nm and 630 nm was observed.

Studying artificial airglow contributes to our understanding of the processes that are realized in the ionosphere as it can be used for the examination of artificial ionization in the perturbed region, the estimation of the role of elastic and inelastic electron collision frequencies, the determination of electric fields, diffusion coefficients and wind velocities of the neutral upper atmosphere, the estimation of the electron energy distribution, etc. [15,16,17].

Below, in Section 2, we describe the geophysical conditions of the experiment, experimental equipment and methods used for data analysis. Section 3 presents the experimental results. In Section 4 the results are discussed. The conclusions are presented in Section 5.

## 2. Experimental Equipment and Methods

For pumping ionosphere, we used the SURA facility situated near Nizhny Novgorod, Russia (geographic coordinates 56.13° N, 46.10° E). During the experiment on 5 August 2024, the pump wave of ordinary polarization (O-mode) radiated vertically from 18:48 UT (LT = UT + 3 h) until 23:28 UT in 6-min cycles: 2.5 min of continuous wave emission followed by a 3.5-min pause. The pump wave frequency $f_{0}$ was chosen to be below the critical frequency of the $F_{2}$ layer, $foF_{2}$. During the time interval considered in the present article, from 19:25 UT to 20:50 UT, $f_{0}$ = 5750 kHz was constant. The effective radiated power $P_{eff}$ of the SURA transmitters was ∼150 MW throughout the experiment. Ionospheric conditions were monitored using ION-FAST ionosonde [18] located in close proximity to the SURA antenna system.

On 5 August 2024, the geomagnetic field was weakly disturbed. On 4 August, a magnetic storm of level G3 (Kp = 7) occurred between 12:00 and 18:00 UT [19]. On 5 August, the magnetic activity decreased to a quiet level (Kp = 3−), but it again reached the threshold of a weak storm of G1 (Kp = 5) after 23:00 UT. According to [20], in the time interval 19–21 UT, the Dst index was ≈−13 (the minimum Dst index on the previous day was −100).

For registration of the airglow the following optical instruments were used at three observation sites.

Directly next to the SURA facility: 3-channel photometer (channels with interference filter transmission centers 391.4 nm, 557.7 nm, and 630 nm with full width at half maximum (FWHM) ∼10 nm, temporal resolution 10 ms and field of view (FoV) ∼12°); CCD camera Andor (filter with a transmission center 557.7 nm and FWHM ∼10 nm) with ∼17° FoV; CCD camera SBig1 with 630 nm interference filter (FWHM ∼10 nm, ∼15° FoV).

Zakluchnaya observation site (55.36° N, 44.33° E, ∼115 km from the SURA facility): similar 630 nm camera SBig2.

Kazan Federal University magnetic observatory (55.56° N, 48.45° E, ∼170 km east of the SURA facility): KEO Sentinel optical system (KEO Scientific Ltd., Calgary, AB, Canada) designed to record the spatial distribution of the 630 nm emission intensity with the interference filter (FWHM ∼2 nm, 145° FoV).

All CCD cameras started acquiring data synchronously at 0 and 30th seconds of each minute with an exposure time 27 s (dead time between frames being 3 s).

Astrometric calibration for cameras with a field of view less than 30 degrees was performed using the Astrometry.net software [21]. Astrometry output data are in the International Celestial Reference System (ICRS). For wide-angle cameras astrometric calibration software developed by us based on the works of [22,23] was used. We implemented an algorithm for automatically identifying bright sources in frames and subsequently correlating with catalog star positions. The software output—azimuth and elevation angle for each pixel in the frame—was converted to the ICRS coordinate system using the Astropy module [24]. For the wide-angle KEO Sentinel frames, azimuths and zenith angles were recalculated for the SURA observation point. The recalculation is performed for a selected layer height above Earth’s surface using the Astropy module [24].

The trends caused by natural variations in nightglow were removed using the method described in the article [16].

## 3. Experimental Results

**Figure 1:**
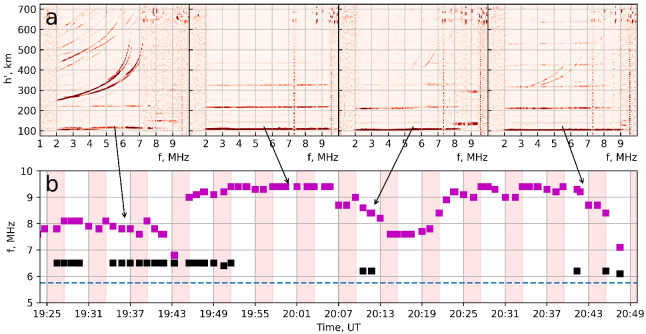
ION-FAST data of $f_{o}F_{2}$ (black), $f_{t}E_{s}$ (pink) and pumping frequency $f_{0}$ (blue dashes) for 5 August 2024 (**b**). Vertical red bars—pump wave turn-on intervals. (**a**)—ionograms for 19:36, 20:00, 20:12 and 20:42 UT (the time is indicated by arrows to (**b**)).

Figure 1 illustrates ionospheric conditions of the 5 August 2024 experiment. In Figure 1b the time course of the F-layer critical frequency ($foF_{2}$, black points) and the sporadic E-layer critical frequency ($f_{t}E_{s}$, violet points) obtained by ION-FAST is shown. Height of the $E_{s}$ layer during the time interval was ∼105–110 km. Moreover, four ionograms registered by ION-FAST at 19:36 UT, 20:00 UT, 20:12 UT and 20:42 UT are inserted in Figure 1a.

It is seen that, during the SURA operation at $f_{0}$ = 5.75 MHz, two types of time intervals with different conditions can be highlighted. There are, first, (i) 19:25–19:31 UT, 19:34–19:53 UT, 20:10–20:13 UT and 20:40–20:49 UT, when translucent $E_{s}$ with $f_{t}E_{s}$ ∼7.8–8.1 MHz (till 9 MHz) does not block the F layer totally, which is well seen in the ionograms together with $E_{s}$ (see Figure 1a). Second, there are intervals (ii) 19:31–19:34 UT, 19:54–20:10 UT and 20:14–20:39 UT, when the F region is totally blocked by the $E_{s}$ with $f_{t}E_{s}$ ∼9.5 MHz, and the pump wave does not penetrate to the F layer.

**Figure 2:**
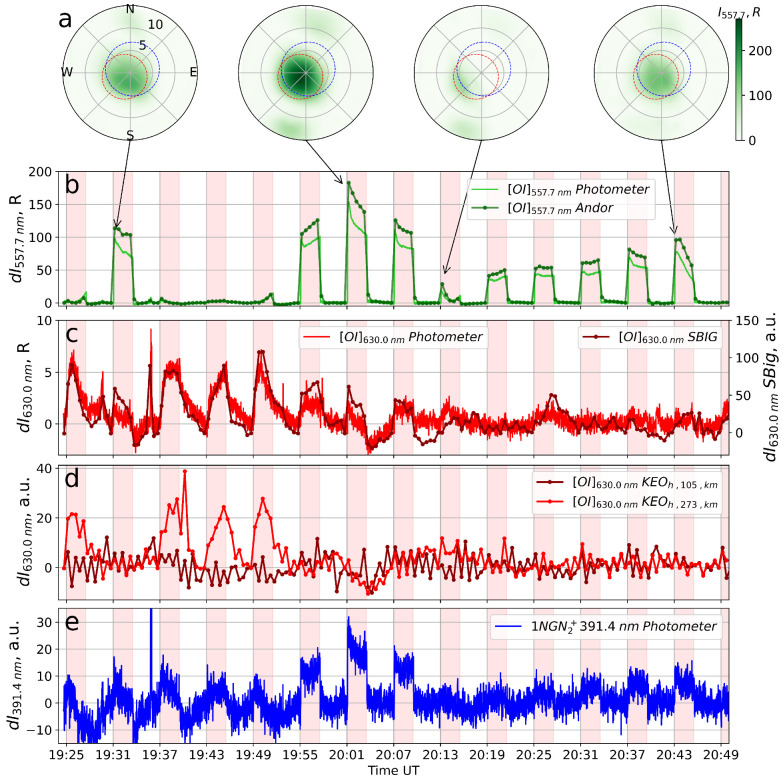
Frames and detrended photometric emission curves on 5 August 2024. (**a**) Andor camera images (557.7 nm) showing the airglow spots. Dashed circles—photometer FoV (blue) and region of max intensity (red). (**b**) Time series of 557.7 nm intensity from photometer (light green) and Andor camera (bright green). (**c**) Same as (**b**) for 630.0 nm (photometer and SBig1). (**d**) KEO Sentinel data mapped to 105 km ($E_{s}$ layer) and 273 km ($F_{2}$ layer) altitudes. (**e**) Photometer data close to 391.4 nm. Vertical red bars—pump wave turn-on intervals. Running averaging with 1 s for photometer data is used.

Figure 2 exhibits results of the airglow measurements. Figure 2a shows frames at 557.7 nm registered by the Andor CCD camera for certain time moments indicated by arrows connecting Figure 2a and Figure 2b. The airglow spots close to the center of the frames are well seen. Additional glow spots, possibly associated with side lobes of the SURA antenna pattern, are also visible in Figure 2a. Figure 2b–e show the detrended photometric curves for the 557.7 (b), 630 nm (c,d) and 391.4 nm (e) lines obtained by the three-channel photometer (solid noisy lines, Figure 2b,c,e), as well as by CCD cameras (curves with dots) Andor (Figure 2b), SBig1 (Figure 2c), and KEO Sentinel (Figure 2d). For the KEO Sentinel camera, a transformation of the FoV for the SURA observation point was performed, taking into account the layer’s height above Earth’s surface. All intensity curves for the cameras are calculated as an average over the FoV in the frame indicated by the red dashed circle in Figure 2a, selected as the region of maximum airglow intensity for the Andor camera. The photometer FoV is shown by the blue dashed circle in Figure 2a. The pumping schedule is indicated by the colored vertical bars.

According to Figure 2c, the dynamics of the red line (630 nm) airglow in the pumping cycles beginning at 19:25, 19:37, 19:43, and 19:49 UT, corresponding to the (i) intervals (translucent $E_{s}$), exhibit typical behavior for pumping the $F_{2}$ ionospheric layer (slow increases and decreases in artificial airglow intensity). Similar but much weaker red line airglow can be distinguished during the cycle beginning at 20:13 UT, also with the translucent $E_{s}$. Using triangulation from the SBig1, SBig2, and KEO Sentinel cameras for cycles with a presence of such typical red line airglow, the altitude of the glow spot observed in these cycles was determined to be ∼273 km.

Figure 2d displays KEO Sentinel data for the altitudes 273 km and 105 km (the latter corresponds to the $E_{s}$ altitude). It is seen that the time course of the red line airglow intensity for the F-region altitude (273 km) is similar for the cycles with translucent $E_{s}$ for SBig1 camera, while no increase is observed in the red line at an altitude of 105 km. During these pumping cycles, a small increase in the green line intensity (about a few Rayleighs) can be distinguished (Figure 2b); the pump-induced blue line airglow was also registered (Figure 2e).

Just after the cycles with the translucent $E_{s}$, in the three subsequent cycles (19:55, 20:01, and 20:07 UT), during the blocking sporadic E, extremely strong artificial airglow (≳100 R) is observed in the green line, and the maximum brightness magnitude across the Andor CCD FoV achieves ∼270 R. Such values were never observed in the previous experiments when artificial green line airglow was associated with the sporadic E layer. Simultaneously, a noticeable enhancement (up to 2 times) was observed for the blue line intensity. Similar enhancements in the green line brightness of the same order are observed during other cycles with blocking $E_{s}$ beginning at 19:31, 20:19, 20:25, 20:31 UT, and with translucent $E_{s}$ in cycles 20:37 and 20:43 UT. Note that, during the latter cycle, the $E_{s}$ and the green line airglow noticeably weaken toward the end of the cycle. The pump-induced blue line is also observed during these cycles but with smaller brightness than during 19:55–20:10 UT.

Note also that, during the cycle 19:31–19:37 UT as well as after 19:55 UT in the data of KEO Sentinel, no increase in the intensity of 630 nm emission is observed either for the F-layer heights or the sporadic E-layer heights of the ionosphere (see Figure 2d). However, unusual behavior of the red line with short development and decay times was observed in cycles beginning at 19:31, 19:55, 20:01, and 20:07 UT, as measured by the photometer (∼0.6 s) and SBig1 camera (see Figure 2c).

## 4. Discussion

The HF-pump-induced airglow generated in the F region of the ionosphere has been studied since the beginning of the ionospheric modification experiments in the early 1970s [2]. For the first time the large 557.7 nm emission produced by HF wave–plasma interactions in the sporadic E layer was observed at the Arecibo heating facility in January 1998 [15]. Later, two successful observations of the green line pump-induced emission occurred at the SURA heating facility [1,16]. In the latter experiment [1], the $E_{s}$-related blue line artificial airglow was also revealed.

In the experiment on 5 August 2024, we obtained some new results on $E_{s}$-related artificial airglow:

1. For the first time at the SURA facility extremely high-intensity (∼270 R) airglow was detected in the 557.7 nm line, associated with a much larger HF pumping $E_{s}$ layer than in previous similar experiments. The emission was observed during the existence of strong blocking $E_{s}$ and half-blocking $E_{s}$ with critical frequency $f_{o}E_{s}$ from 7.8 to 9.5 MHz. The pump wave frequency used was $f_{0}$ = 5.75 MHz. In previous experiments the sporadic E layer was half-blocking; the maximum brightness of the pump-induced $E_{s}$-related airglow was 55 R for the pump wave frequency $f_{0}$ = 3.175 MHz and critical frequency $f_{o}E_{s}$ ∼4.5 MHz [15], ∼10 R for $f_{0}$ = 4.3 MHz and $f_{o}E_{s}$ ∼7 MHz [16], and ∼7 R for $f_{0}$ = 5.32 MHz and $f_{o}E_{s}$ ∼5.6 MHz [1].
  In parallel with the airglow spot attributed to the main lobe of the SURA antenna pattern, two weaker glow spots of lower intensity in the green line in the southwest and northeast directions (∼12° zenith angles) corresponding to the side lobes of the antenna pattern were detected.
2. During the cycles with strong green line airglow (19:31, 19:55, 20:01, and 20:07 UT), there was unusual temporal behavior of the red line emission with sharp onset of increase and fast decay after the pump wave switch on/off (see Figure 2c).
3. Similar to [1], a pump-induced enhancement in the blue band airglow was seen during the $E_{s}$ existence. The enhancements were observed both for blocking $E_{s}$ (large brightness, simultaneously with strong green line emission) and for partially blocking $E_{s}$, with more moderate brightness, of the same order as in the experiment of [1].

Now it is generally accepted that the enhancement of the airglow brightness in all the lines under investigation in this paper is a consequence of the excitation of the ionospheric gases (neutral and ions) by the impact of electrons accelerated by pump wave parametrically induced plasma waves.

The airglow is considered to be evidence that the HF-modified electron distribution function is non-Maxwellian because a significant flux of suprathermal electrons is required to produce it. This is confirmed by a number of papers that considered theory and computer modeling of electron acceleration, electron ohmic heating, optical emission and additional ionization due to particle energization and a comparison of the results with the data of specific experiments [14,25,26]. These papers focused on F-layer pump-induced phenomena. However, the physical explanation of the observed F-layer phenomena could not be considered as totally complete.

Due to the small amount of experiments [1,15,16] the understanding of the observed $E_{s}$-layer airglow features is quite limited. Particularly, in [15], it is shown that the pump power at the $E_{s}$ altitude exceeded the threshold of the parametric instabilities; Refs. [16,27] discussed applications of the obtained results to the diagnostics of $E_{s}$ peculiarities (wind velocity and visualizing a horizontal $E_{s}$ structure). Ref. [17] showed that ohmic heating is not sufficient to provide strong enhancement in the Arecibo experiment [15].

The most impressive result obtained on 5 August 2024 in our experiment is the extremely large intensity of the green line pump-induced airglow, exceeding one obtained at Arecibo by 5 times and one in previous SURA experiments by 25 and 40 times for the pump wave power of the same order. This means, most probably, that the obtained large intensity value is determined, first of all, by features of the $E_{s}$ layer that occurred during our experiment. The most noticeable difference is a much larger critical frequency $f_{t}E_{s}$ and the long duration of the layer’s existence. It is difficult to establish the reason for this. The essential point to mention is that the experiment was performed a day after a rather strong magnetic storm occurred. The results of [28] show that E layers can be significantly enhanced during the recovery phase of a geomagnetic storm.

The other point is that, in August, the Perseid meteor shower takes place, a phenomenon that occurs when Earth passes through a stream of dust particles left behind by Comet Swift–Tuttle. In [29] it is shown that the marked seasonal dependence of sporadic E correlates well with annual variations in sporadic meteor deposition in the upper atmosphere.

Unfortunately, we are not familiar with the details of the models of sporadic layer appearance in different geophysical conditions; this point should be deeply studied. Notice that a large amplification of artificial airglow was obtained only in the green line; the intensity in the blue line was approximately of the same order as in [1].

**Figure 3:**
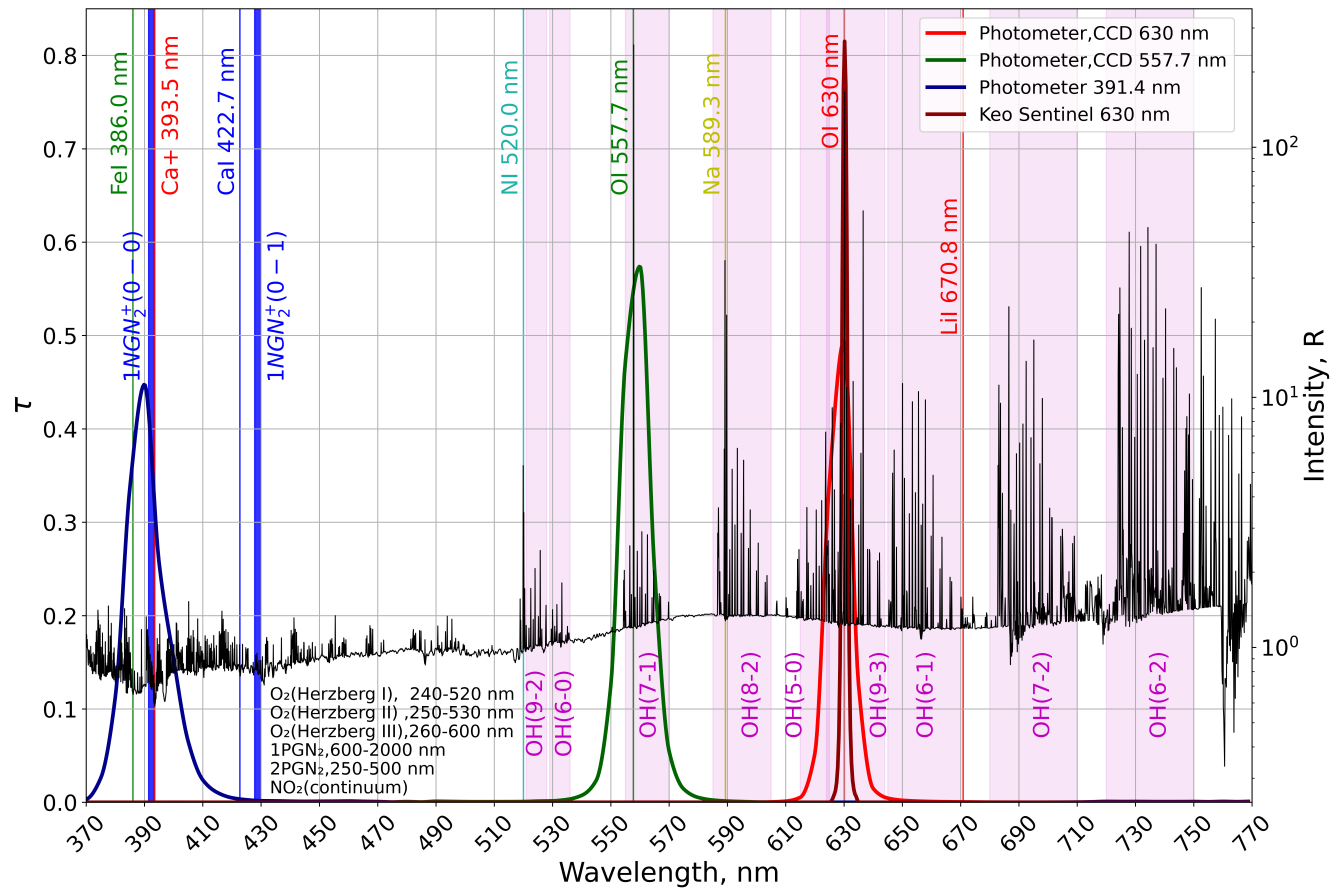
Spectral transmittance τ of the filters and the airglow spectrum in the interval 370–770 nm.

For further discussion, Figure 3 demonstrates spectral transmittance τ of the optical equipment filters and the natural airglow spectrum in the wavelength interval 370–770 nm, borrowed from [30].

The unusual behavior of the red line with sharp rise and fall fronts in intensity in cycles beginning at 19:55, 20:01, and 20:07 UT, as measured by the photometer and SBig1 camera (see Figure 2c), can be due to induced emission in the hydroxyl bands OH(9-3) and OH(5-0) with radiative lifetime $\tau <6\cdot 10^{-2}$ s [31]. The photometer and SBig1 camera are more efficient in recording these bands than KEO Sentinel due to their larger FWHM ∼10 nm (see Figure 3). The KEO Sentinel optical system is equipped with a filter with FWHM ∼2 nm (see Figure 3).

The detection of hydroxyl emission during the recording of 630 nm emission was repeatedly observed. For example, in the work [32], during observations with a limb instrument LiVHySI (effective spectral bandwidth ∼22 nm), two distinct layers of airglow near a wavelength of 630 nm were detected. The upper $O{(}^{1}D)$ layer covers an altitude range of 200–300 km, and the lower thin OH(9-3) layer is limited to an altitude range of 80–100 km. Airglow of OH and $O{(}^{1}D)$ emissions was also recorded during limb measurements by the ISUAL (Imager of Sprites and Upper Atmospheric Lightning) instrument on board the FORMOSAT 2 satellite [33]. The measurements were carried out using a CCD camera at a wavelength of 630 nm with an FWHM of ∼7 nm.

In [15] artificial airglow in the spectral range of 640–680 nm, as well as an increase in intensity in the spectral range of 710–760 nm during HF pumping of the $E_{s}$ layer, were noted. The authors associated the former with the emission of the first positive band of $N_{2}$, which required significant electron fluxes at energies ≥9 eV. The authors did not associate the increase in intensity in the latter range with specific atmospheric emissions. According to [34], OH(6-1) and OH(7-2) hydroxyl bands also emit in spectral ranges of 640–680 nm and 710–760 nm (see Figure 3). It is possible that the authors of [15] recorded an increase in the intensity of these bands.

Further, in [35], apparent inconsistencies in the theoretical cross sections and reaction rates were found, indicating that additional measurements of electron-impact excitation of OH are needed. In [36] it was found that energetic electron precipitation has a small effect on the production rate of $OH^{*}$-excited vibrational states. However, the production rate increases drastically when geomagnetic activity increases. Therefore we conclude that further research into the excitation of OH emission by impact is needed.

The increase in intensity in the blue channel of the 391.4 nm photometer can be due to the following factors:

- Excitation and subsequent emission in the $1NGN_{2}^{+}$ band (391.4 nm). This scenario is the most plausible provided that there is a sufficient concentration of $N_{2}^{+}$ present at the altitude of the $E_{s}$ layer. The energy required to excite an existing ion $N_{2}^{+}$ from the ground state is ≈3.17 eV [37]. By electron impact the excited $N_{2}^{+}$ is often formed directly from the neutral $N_{2}$. In this case, the energy of $N_{2}$ ionization is ≈15.58 eV [37,38], and it will be summed with the excitation energy. In total, this comprises ≈18.75 eV.
- Emission of metals FeI 386.0 nm and $Ca^{+}$ 393.5 nm, which also fall within the passband of the 391.4 nm photometer filter (see Figure 3) [34]. The excitation energies are 3.2 eV and 3.151 eV, respectively [39].

## 5. Conclusions

Experimental observations of artificial airglow of the ionosphere at $E_{s}$-layer altitudes induced by powerful HF radiation from heating facilities are extremely limited. On 5 August 2024, several new features of artificial airglow behavior were obtained:

- For the first time the extremely high intensity (∼270 R) of the artificial airglow in the 557.7 nm line, associated with the effect on the $E_{s}$ layer, was detected at the SURA facility. The detection of additional glow spots of lower intensity in the green line in the southwest and northeast directions from the main spot (∼12° zenith angle; see Figure 2a) may be associated with the side lobes of the SURA antenna pattern.
- The behavior of the red line with sharp fronts of increase and decrease in intensity in the heating cycles started at 19:31, 19:55, 20:01, and 20:07 UT, much faster in comparison with the intensity growth and decay of the $O{(}^{1}D)$ response typical for generation in the F region, in the cycles started at 19:37, 19:43, and 19:49 UT (see Figure 2c), which may be associated with artificial airglow in the hydroxyl bands, presumably OH(9-3) and OH(5-0).
- The intensity increase in the blue channel of the 391.4 nm photometer may be caused by excitation of the $1NGN_{2}^{+}$ band (391.4 nm), FeI emission line (386.7 nm), $Ca^{+}$ emission line (393.5 nm) or a combination of them.

For further detailed analysis, it is desirable to conduct experimental campaigns to detect artificial airglow using spectrometric equipment and under various geophysical conditions.

## Data Availability

Dataset available on request from the authors.

## Abbreviations

The following abbreviations are used in this manuscript:

CCD	Charge-Coupled Device
EISCAT	European Incoherent Scatter Scientific Association
FoV	Field of View
HAARP	High-Frequency Active Auroral Research Program
HF	High Frequency
